# A Review of *Canarii Fructus* (*Canarium album*) Polyphenols: From Efficient Extraction to Mechanistic Understanding and Functional Food Development

**DOI:** 10.3390/foods15132410

**Published:** 2026-07-07

**Authors:** Jie Ma, Rongqing Yang, Ziqiao Xu, Haonan Zhang, Baozhong Duan, Haizhu Zhang, Fumei He, Yongcheng Yang, Xubing Chen, Conglong Xia

**Affiliations:** 1 College of Pharmacy, Dali University, Dali 671003, China; 15187369453@163.com (J.M.); 15368660693@163.com (R.Y.); xzq578440737@163.com (Z.X.); zhn2504612109@gmail.com (H.Z.); bzduan@126.com (B.D.); hzningjing@163.com (H.Z.); hefumei2023@126.com (F.H.); chenxubing@dali.edu.cn (X.C.); long7484@126.com (C.X.); 2 Yunnan International Joint Laboratory of Characteristic Medicinal and Edible Resources, Dali 671003, China

**Keywords:** *Canarii Fructus* polyphenols, extraction technologies, bioactivity, multi-target mechanisms, functional food

## Abstract

*Canarii Fructus* (*Canarium album*) is a rich source of polyphenols with significant potential for functional food applications. This review summarizes recent advances in the composition, extraction technologies, biological activities, and utilization prospects of *Canarii Fructus* polyphenols (CFPs). More than 30 polyphenolic compounds have been identified, with gallic acid and ellagic acid as the major constituents, accounting for approximately 38.8% and 14.3% of the total phenolics, respectively. The fruit contains about 300 mg/100 g fresh weight of phenolic compounds. Emerging extraction technologies, including ultrasound-assisted extraction, microwave-assisted extraction, and ultrasound–microwave-assisted extraction (UMAE), have improved extraction efficiency, with UMAE achieving yields of up to 6.33% within 4.4 min. Unlike previous studies focusing primarily on phytochemical characterization or pharmacological activities, this review provides a comprehensive food-oriented perspective by integrating chemical diversity, extraction strategies, molecular mechanisms, bioavailability challenges, and functional food applications of CFPs. CFPs exhibit antioxidant, anti-inflammatory, antiviral, antibacterial, antitumor, anti-aging, hepatoprotective, and metabolic regulatory activities through pathways including Nrf2/ARE, NF-κB, PI3K/Akt, and AMPK. Representative bioactivities include α-glucosidase inhibition (IC_50_ = 9.914 × 10^−3^ μg/mL) and regulation of lipid metabolism via AMPK activation. Particular attention is given to emerging approaches, including green extraction technologies, nanodelivery systems, and AI-assisted target discovery. Current limitations related to low bioavailability, unclear structure–activity relationships, and insufficient in vivo evidence are also discussed. Overall, CFPs represent a promising natural resource for the development of functional foods and nutraceuticals.

## 1. Introduction

**Figure 1:**
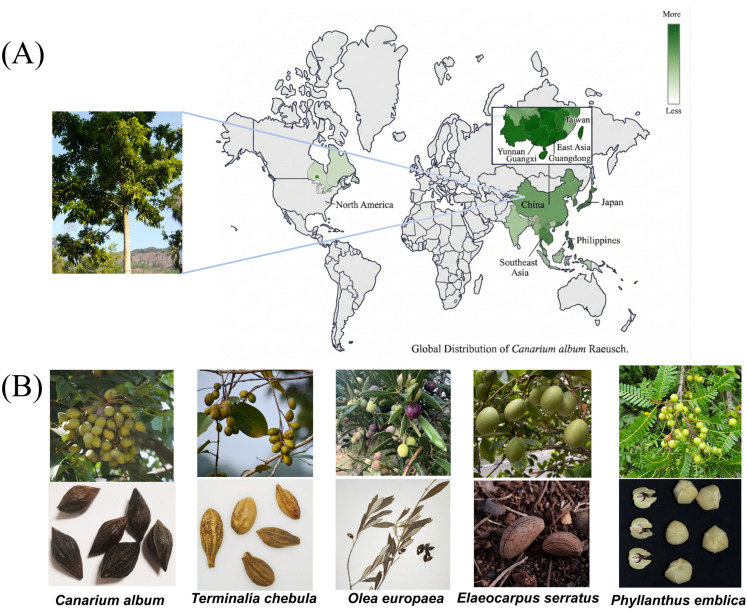
(**A**) Global distribution map of CF. (**B**) Five easily confused varieties of olive. All images in Figure 1B are sourced from the free web: www.inaturalist.org (accessed on 18 June 2026).

*Canarii Fructus* (CF), the mature fruit of *Canarium album* (Lour.) Raeusch, a member of the Burseraceae family, is a well-known tropical and subtropical fruit crop [1]. China is the largest producing area of Chinese olive, especially Fujian Province, its main origin, with over 140,000 acres of planting area. In addition to China, Chinese olive is cultivated in Vietnam, Japan, Laos, Myanmar, Thailand, the Philippines, Malaysia, Indonesia, India, and Sri Lanka, encompassing almost the entirety of Asia (Figure 1A) [2]. *C. album* (CA) is a long-cultivated medicinal fruit tree and an economically important crop in East Asia, where its cultivation and utilization are closely tied to traditional practices [3]. With the rising demand in the health-oriented food industry, research interest has increasingly focused on improving the preservation, processing, and utilization of CF [4], as well as elucidating its bioactive constituents.

CF exhibits multiple pharmacological effects, including antibacterial [5] and anti-inflammatory effects [6], antioxidant activity [7], and anticancer properties [8], as well as antiviral effects [9]. Its chemical constituents are complex and diverse, primarily comprising volatile oils, flavonoids, polysaccharides, polyphenols, terpenoids, amino acids, and mineral elements [10], among which polyphenolic substances are not only one of the most abundant and active components in CF but also serve as core indicators influencing its flavor and functional quality [11].

Despite increasing interest in CA and its bioactive constituents, existing reviews have primarily focused on traditional medicinal uses, general phytochemical composition, or specific pharmacological activities [10], and no systematic review has yet approached CFPs from a food science perspective. The relationships among phenolic composition, extraction technologies, molecular mechanisms, bioavailability, and food applications remain fragmented across the literature, while emerging topics such as green extraction, nanodelivery systems, AI-assisted target discovery, and translational challenges in functional food development have not been comprehensively discussed. To fill these gaps, this review provides a food-oriented synthesis that integrates chemical diversity, extraction and purification techniques, biological activities, molecular mechanisms, bioavailability constraints, and future functional food applications, aiming to establish a framework for sustainable utilization and value-added development of CFPs (Table 1).

Compared with well-established polyphenol sources like tea (rich in catechins such as EGCG) and grapes (containing anthocyanins, proanthocyanidins, and resveratrol), both extensively studied and widely applied for their antioxidant, anti-inflammatory, and cardiometabolic benefits [12,13], CFPs are still at an early research stage. They feature a higher proportion of hydrolysable tannins and diverse phenolic acids, yet systematic understanding of their structure–activity relationships and industrial applications remains limited. Recent dietary polyphenol reviews further emphasize that despite rapid progress in tea and grape research, challenges persist in bioavailability, metabolism, and food system interactions, underscoring the need to explore alternative polyphenol-rich resources like CFPs [14,15]. Thus, CFPs represent a complementary and underutilized functional food resource with a distinctive chemical profile and considerable potential for application in health-oriented food systems.

To ensure the comprehensiveness and reliability of this review, a systematic literature search was conducted across multiple international and Chinese databases, including Web of Science, Scopus, PubMed, ScienceDirect, Google Scholar, and China National Knowledge Infrastructure (CNKI). The literature retrieval was performed using a combination of English and Chinese keywords, including “*Canarium album*”, “Chinese olive”, “*Canarii Fructus*”, “CFP”, “polyphenols”, “phenolic compounds”, “flavonoids”, “extraction”, “biological activity”, and “functional food”, as well as the Chinese terms “青果多酚” and “橄榄多酚”.

The search period covered publications from 2000 to 2025 to capture both foundational studies and the most recent advances in CFP research. Only peer-reviewed original research articles and review papers written in English or Chinese were considered. Studies unrelated to CFPs or lacking sufficient experimental or compositional information were excluded. Duplicate records, conference abstracts without full data, and non-peer-reviewed sources were also removed during screening. The final selection was based on relevance to CFP chemical composition, extraction technologies, biological activities, food applications, and safety evaluation.

## 2. Botanical Characteristics and Identification of CF

### 2.1. Morphology and Geographical Distribution of CF

As recorded in the Chinese Pharmacopoeia, *C. Fructus*, the dried mature fruit of *Canarium album* Raeusch. (family Burseraceae), commonly known as Chinese olive, white olive, or sweet olive, is characterized by a neutral nature and a sweet–sour taste. Traditionally, it is used to clear heat and detoxify the body, soothe the throat, promote saliva production, and alleviate conditions such as sore throat, cough with sticky phlegm, vexation and thirst, and poisoning from fish or crab. As a medicinal resource with a long history, CF originated in China and has been cultivated for more than 2000 years. Botanically, the fruit is ovoid to fusiform, measuring 2.5–3.5 cm in length, smooth, and yellow-green in color (Figure 1B). The Chinese olive kernel is spindle-shaped with longitudinal cracks, and there are seeds in each of the three chambers, rich in oil that can be used for edible oil extraction [16]. The exocarp becomes thick and wrinkled upon drying, while the endocarp tapers into a pointed shape. The pulp exhibits a distinctly bitter and astringent taste, followed by a mild sweetness after prolonged chewing, which contributes to its characteristic sensory profile [17].

### 2.2. Factors Affecting CFP Composition

Current evidence indicates that cultivar and fruit maturity are among the most important factors influencing the composition and accumulation of CFPs. Significant differences in phenolic profiles have been reported among CA cultivars. For example, the cultivar “Tanxiang” accumulates relatively high levels of ellagic acid, corilagin, and related phenolics, which has been associated with elevated expression of key genes involved in the shikimate pathway, whereas low-astringency cultivars such as “Makeng 22” generally exhibit lower phenolic contents due to reduced phenylpropanoid metabolism and enhanced polyphenol oxidase activity [18,19,20]. Fruit developmental stage also exerts a pronounced influence on CFP accumulation. Total phenolic content typically follows a dynamic pattern, increasing during early fruit development, reaching a maximum approximately 102 days after flowering, and subsequently declining during fruit ripening and softening [21].

The effects of geographical origin and climatic conditions remain less well understood. Existing studies have documented morphological and adaptive differentiation among CA germplasm resources distributed across southern China, suggesting that geographic isolation may indirectly influence phenolic metabolism through genetic variation. In addition, environmental factors such as light intensity, temperature, and sun exposure stress are recognized regulators of phenolic biosynthesis in many plant species and may similarly affect CFP accumulation. However, systematic comparative studies examining the independent contributions of geographical and climatic factors to CFP composition are currently lacking. Future research integrating metabolomics, genomics, and environmental data will be essential for elucidating these relationships and establishing robust quality control standards for CFP-rich raw materials.

### 2.3. Differentiation of CF from Morphologically Similar Species

CF exhibits certain morphological similarities to *Chebulae Fructus Immaturus* (also referred to as Tibetan olive or Terminalia chebula fruit), *Olea europaea* L. (olive), *Elaeocarpus serratus* L. (Ceylon olive), and *Phyllanthus emblica* L. (Yunnan olive or emblic). However, despite their comparable external appearance, these species differ markedly in taxonomic origin and should not be used interchangeably. Accurate differentiation is therefore essential for both practical applications and scientific research. *Chebulae Fructus Immaturus* is the dried immature fruit of *Terminalia chebula* Retz. (family Combretaceae). It is oblong–ovoid in shape and shows distinct differences from CF in pericarp epidermal cell morphology, which provides a reliable microscopic basis for authentication [17]. *Olea europaea*, belonging to the genus Olea within the family Oleaceae, is primarily distributed along the Mediterranean basin and was only introduced into China for cultivation during the 20th century [22]. *Elaeocarpus serratus*, a member of the family Elaeocarpaceae, originated in India and surrounding regions and was similarly introduced into China in the 20th century [23]. *Phyllanthus emblica*, belonging to the family Euphorbiaceae and commonly known as emblic or amla, is widely distributed across subtropical and tropical areas of China, India, Indonesia, and the Malay Peninsula (Figure 1B) [24].

### 2.4. Importance of Species Authentication for CFP Research

Accurate species authentication is a prerequisite for reliable phytochemical and biological investigations. Although several fruits exhibit morphological similarities to CF, their taxonomic origins and chemical compositions differ substantially. Misidentification may lead to significant variations in polyphenolic profiles, resulting in inconsistent data regarding compound characterization, extraction efficiency, antioxidant capacity, and other biological activities. Such discrepancies can compromise the reproducibility and comparability of experimental findings across studies. Furthermore, accurate authentication is essential for establishing quality control standards, evaluating structure–activity relationships, and ensuring the safety and efficacy of CFP-derived functional food ingredients. Therefore, rigorous botanical identification should be considered a fundamental step in CFP research and industrial utilization.

## 3. Advances in CFPs

### 3.1. Structural Classification and Chemical Diversity of CFPs

Polyphenols are widely present in plant-derived foods, particularly abundant in fruits, vegetables, grains, and medicinal plants, and have demonstrated significant application potential in the functional food field due to their unique chemical structures and broad biological activities [15]. CF exhibits remarkably high phenolic content, ranking first among 68 Chinese medicinal materials, with levels reaching 300 mg/100 g fresh weight, higher than those of common fruits such as grapes and apples, highlighting its important value as a natural source of polyphenols [25]. Early studies have identified multiple key polyphenolic compounds in CF, with total phenolic content determined to be approximately 1.5%. Among these, gallic acid and ellagic acid showed the most prominent relative contents, reaching 38.8% and 14.3%, respectively [26]. To date, over 30 polyphenolic compounds have been identified from CF (Table 2).

#### 3.1.1. Comparison and Optimization of Assay Methods for Polyphenols

In the extraction and quality analysis of polyphenols from CF, establishing an accurate and reliable method for content determination is crucial. Commonly used methods include the ferric tartrate method, the Folin–Ciocalteu reagent method, and UV spectrophotometry. Xiang et al. [47] initially established a determination system based on the Folin–Ciocalteu method: add 1.5 mL of Folin–Ciocalteu reagent and 6 mL of 10% sodium carbonate solution, react at 30 °C for 60 min, and measure at 765 nm.

To evaluate the applicability of the three methods, Xie et al. [21] conducted a systematic comparative study. In terms of sensitivity, the order from low to high limits of detection and quantification was UV spectrophotometry < Folin–Ciocalteu method < ferric tartrate method, with significant differences (*p* < 0.05). In terms of accuracy, the order from high to low recovery rates was ferric tartrate method > Folin–Ciocalteu method > UV spectrophotometry, but the differences were not significant (*p* > 0.05). All three methods showed good repeatability. Regarding the measured content, the values obtained by the Folin–Ciocalteu method and UV spectrophotometry were significantly higher than those obtained by the ferric tartrate method (*p* < 0.01); the Folin–Ciocalteu method gave slightly higher values than UV spectrophotometry, but the difference was not significant (*p* > 0.05). Further analysis showed good linear correlations between the results of any two of the three methods.

In a comprehensive comparison, although the Folin–Ciocalteu method offers both good sensitivity and accuracy, UV spectrophotometry is simpler to operate, lower in cost, and highly correlated with the Folin–Ciocalteu method, making it an ideal and practical method for the determination of polyphenol content in Chinese olive.

Additionally, high-performance liquid chromatography coupled with mass spectrometry (HPLC–MS) has become one of the most powerful analytical tools for the characterization of CFPs due to its high sensitivity, selectivity, and structural elucidation capability [6]. Techniques such as HPLC-DAD-ESI-MS, UPLC-MS/MS, and UPLC-Q-Exactive Orbitrap/MS have been widely applied to identify and quantify phenolic acids, hydrolysable tannins, and flavonoids in CA [48]. For example, HPLC-DAD-ESI-MS enabled the identification of gallic acid, ellagic acid, methyl gallate, ethyl gallate, and several HHDP derivatives in CF, whereas UPLC-Q-Exactive Orbitrap/MS has recently been used to characterize more than 40 phenolic compounds across different cultivars [39,49]. These advanced analytical platforms provide essential support for phytochemical profiling, quality control, metabolomic studies, and the identification of potential marker compounds for CFP standardization.

#### 3.1.2. Potential Marker Compounds for CFP Standardization

Given the substantial variability in CFP composition caused by cultivar, maturity stage, geographical origin, and processing conditions, the establishment of suitable marker compounds is essential for quality control and standardization. According to current marker selection principles, ideal phytochemical markers should exhibit high abundance, frequent occurrence, chemical stability, analytical accessibility, and biological relevance [50].

Based on the phytochemical evidence summarized in this review, gallic acid and ellagic acid are the most promising primary marker compounds for CFP standardization. These compounds are consistently detected across independent studies, represent the predominant phenolic constituents of CF, and possess well-documented antioxidant, anti-inflammatory, and metabolic regulatory activities. Chlorogenic acid may serve as a complementary marker because of its frequent occurrence and recognized role as a quality control marker in several polyphenol-rich botanical products. In addition, corilagin, quercetin, and kaempferol derivatives may function as secondary markers reflecting the characteristic tannin and flavonoid composition of CFPs [51,52,53].

Future studies integrating metabolomics, chemometric analysis, and bioactivity-guided screening are warranted to establish CFP-specific quality markers (Q-markers) and develop standardized chemical fingerprints for functional food and nutraceutical applications [52].

### 3.2. Extraction and Separation Technologies for CFPs

**Figure 2:**
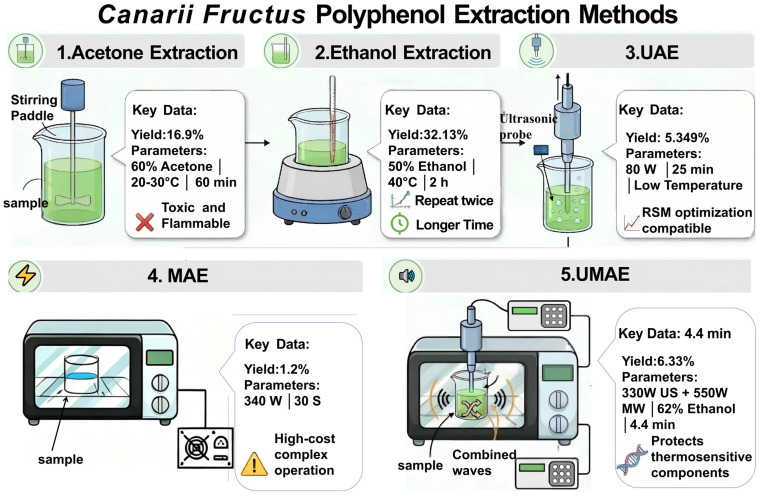
Extraction methods. Traditional solvent extraction (60% aqueous acetone, stirring for 60 min, yield 16.9%, but limited by solvent toxicity) and its improved conditions, compared with three modern extraction technique, including ultrasound-assisted extraction (UAE, yield 5.349%), microwave-assisted extraction (MAE, yield 1.2%), and Ultrasound–Microwave Synergistic Extraction (UMAE, yield 6.33%, only 4.4 min required)—showing their process conditions, yields, advantages, and disadvantages.

Due to the abundance of hydroxyl groups, polyphenolic substances are generally water-soluble and readily soluble in polar organic solvents such as methanol, ethanol, and acetone. Therefore, water, ethanol, or acetone solutions are mostly employed as extraction solvents for CFPs, with extraction temperatures typically ranging between 20 and 72 °C. Based on the auxiliary techniques employed during the extraction process, extraction methods for CFPs can be broadly categorized into five types (Figure 2).

#### 3.2.1. Traditional Extraction

In the traditional field of polyphenol extraction, stirring-assisted extraction (e.g., Mechanical Stirring Extraction, MSE) and maceration (also known as soaking) are two fundamental and widely applied solid–liquid extraction techniques. Maceration relies on natural diffusion, offering simple operation and low equipment requirements, making it suitable for protecting heat-sensitive components (e.g., recommended for heat-sensitive pharmaceutical preparations) [54]. However, it is time-consuming (e.g., blackthorn requires 84 days of maceration to achieve maximum polyphenol content) [55]. Moreover, in olive oil wastewater, maceration yields significantly higher contents of total phenols, flavonoids, and tannins, as well as greater antioxidant activity (FRAP/DPPH/ABTS), compared to liquid–liquid extraction [56]. Stirring-assisted extraction (e.g., MSE) actively enhances mass transfer, greatly reducing extraction time (e.g., 15 min for orange waste, 5 min for spinach) [56]. In certain matrices, the polyphenol yield from MSE is 1–2 times higher than that from ultrasound-assisted extraction, with a notable increase in antioxidant activity. Microwave-stirring coupling (MW-IA) further optimizes extraction kinetics, reaching equilibrium within 16 min and achieving the highest phenolic recovery [57]. Under the conditions of a solid-to-liquid ratio of 1:20 in 60–70% aqueous acetone solution, with stirring at 20–30 °C for 60 min, the extraction yield can reach 16.9% [26]. However, the toxicity and flammability of acetone constrain its scalability and industrial applicability. After comparative extraction experiments, it was concluded that the optimal solvent for extracting polyphenols from Chinese olives using the solvent extraction method is 50% ethanol, with an optimal extraction time of 2 h, a temperature of 40 °C, a solid-to-liquid ratio of 1:30, and two extraction cycles (Table 3).

#### 3.2.2. Emerging Extraction Technologies

Ultrasound-assisted extraction (UAE) utilizes cavitation effects and other ultrasonic actions to accelerate the extraction process. Ref. [26] found that ultrasonic extraction at 80 W power for 25 min achieved significantly higher efficiency than traditional maceration, with comparable extraction yields. The process is often optimized using Plackett–Burman design combined with response surface methodology, showing potential applications in natural polyphenol extraction [61] and demonstrating superiority in existing studies. Through response surface optimization, Liu determined that under optimal conditions, including an ultrasonic power of 186 W, an ethanol concentration of 50%, an extraction time of 22 min, and a solid-to-liquid ratio of 1:14, the yield of CFPs could reach 5.349% [62] (Table 4).

Microwave-assisted extraction (MAE) relies on electromagnetic wave heating, characterized by strong penetration and uniform heating. The authors of [26] reported polyphenol extraction at 340 W power for 30 s, but with a yield of only 1.2%. However, response surface methodology is also applicable for optimizing such processes to maximize polyphenol yield and biological activity [65].

The ultrasound-microwave synergistic-assisted extraction (UMAE) method, combining the advantages of both ultrasound and microwave, demonstrates higher efficiency and was preliminarily established and further optimized through response surface methodology. Synergistic extraction with 62% ethanol, ultrasonic power of 330 W, and microwave power of 550 W for 4.4 min could achieve a yield of 6.33% [26,66]. This synergistic technique can reduce the degradation of heat-sensitive polyphenols and has also been confirmed to significantly enhance total phenolic content and antioxidant activity in the extraction of other plant active components, such as red onion anthocyanins and Dictyophora polysaccharides [67,68].

Additionally, subcritical water extraction, as an environmentally friendly technology, has also been applied. Ref. [69] explored the extraction process for CFPs, specifically extracting with water under conditions of 5–10 MPa pressure and 200–270 °C temperature for 20–60 min.

It should be noted that direct comparison of extraction yields reported in different studies remains challenging. Extraction efficiency is influenced by multiple factors beyond the extraction technology itself, including cultivar, fruit maturity, geographical origin, drying and pretreatment methods, particle size, solvent type and concentration, solid-to-liquid ratio, extraction temperature, extraction time, and analytical procedure. Furthermore, extraction yields are often expressed using different calculation methods and target parameters, such as crude extract yield, total phenolic yield, or recovery of individual compounds. Consequently, variations observed among studies may reflect differences in experimental design rather than the intrinsic superiority of a particular extraction technique. Therefore, the extraction yields summarized in this review should be interpreted as representative results obtained under specific conditions rather than directly comparable performance indicators. Future investigations employing standardized raw materials, harmonized extraction protocols, and unified reporting criteria will be essential for establishing meaningful benchmarks and accurately evaluating the relative advantages of different CFP extraction technologies.

### 3.3. Future Perspectives: Toward Green and Intensified Extraction Strategies

Future research on CFP extraction is expected to advance towards greener, more efficient and more intelligent approaches. Recently developed innovative extraction techniques have gained attention for their environmental advantages, including reduced solvent consumption, shorter processing time and improved extraction yield and quality, which help address the drawbacks of conventional methods [70]. These emerging techniques are increasingly recognized for their capacity to enhance the overall yield and selectivity of plant-derived bioactive compounds [71].

Key research priorities include establishing green extraction processes, expanding the application of CFPs in high-value sectors such as functional foods and antiviral formulations, and improving the utilization of by-products to enhance the economic and environmental sustainability of the industrial chain. In terms of process optimization, artificial intelligence tools can be incorporated. Artificial neural networks have been successfully used to predict optimal extraction parameters for bioactive components in pumpkin, demonstrating their value in evaluating parameter significance and guiding process optimization [72]. In addition, emerging green technologies suitable for large-scale production, such as high-voltage pulsed electric fields, warrant further investigation [73]. This technology does not cause color degradation or reduced antioxidant capacity in heat-sensitive polyphenols such as anthocyanins [74]. It can reduce the degradation of heat-sensitive polyphenols with retention rates of 50–80% [75] and can minimize the degradation of chemical structures and activity loss caused by temperature, pH, and light during food processing and storage when used as natural antioxidants to delay food oxidation [76].

Regarding extraction methodologies, enzyme-assisted extraction remains a promising direction. The combined use of cellulase to hydrolyze cell wall polysaccharides has been shown to substantially increase polyphenol yields [77]. This approach holds potential for CFP extraction and may support their future development and industrial application within the food sector.

Green and efficient extraction of plant polyphenols represents a critical pathway for the valorization of agricultural by-products and the realization of a circular bioeconomy. Current technological systems can be categorized into two synergistic strategies: one is physical intensification technologies represented by high-intensity ultrasound-assisted extraction (HIUAE), pulsed electric field (PEF), and Pressurized Liquid Extraction (PLE); the other is green solvent systems centered on Deep Eutectic Solvents (DESs) and Natural Deep Eutectic Solvents (NADESs). These two approaches are often coupled to achieve synergistic efficiency enhancement.

High-Intensity Ultrasound-Assisted Extraction (HIUAE) is a green non-thermal extraction technology that leverages ultrasonic cavitation, acoustic streaming, and micro-jetting effects to enhance cell disruption and mass transfer, significantly improving the release efficiency of bioactive components while effectively preserving heat-labile structures. Notably, the recent literature often regards HIUAE as an intensified form of UAE rather than a completely independent technology. Many studies use only the term “UAE”, yet their experimental conditions (typically frequency 20–40 kHz, power density >1 W/cm^2^, amplitude 60–100%) already fall within the HIUAE category. In recent years, HIUAE has been successfully applied to extract polyphenols from various plant sources. For example, the recovery rates of phenolic acids and flavonoids from grape seeds, pomegranate peels, tea leaves, and rosemary are generally superior to those of conventional solvent extraction [78].

Pulsed electric field (PEF) is a non-thermal, green physical processing technology that applies short-duration high-voltage pulses (typically 0.1–10 kV/cm) to induce electroporation of cell membranes, forming transient micropores, which significantly enhances cell permeability and mass transfer efficiency, thereby efficiently releasing intracellular bioactive compounds while avoiding thermal degradation. Using PEF, the total polyphenol extraction yield from olive leaf waste increased by 38% and specific metabolites by up to 117% [79]. Extraction of phenolics from whole dragon fruit increased yield by 135% and DPPH activity by 145%; epigallocatechin gallate EGCG,799.9 mg/g, was detected for the first time in Stelechocarpus burahol leaves, and epicatechin increased from 338.2 to 921.4 mg/g [80].

Pressurized Liquid Extraction (PLE), also known as Accelerated Solvent Extraction (ASE), is a green extraction technology that maintains the solvent in a liquid state under high-temperature and high-pressure conditions, thereby enhancing mass transfer efficiency. Its core principle involves increasing temperature (typically 60–200 °C) and pressure (typically 5–15 MPa), which reduces solvent viscosity and enhances diffusion capacity, promoting efficient release of target compounds from the matrix while significantly shortening extraction time and reducing solvent consumption [81]. In pomegranate pomace, the phenolic yield by PLE of 11.96 mgGAE/g was significantly higher than that of stirred liquid extraction and UAE, while the protein retention of the residual biomass was higher [82]. Under conditions of 122 °C and 17 min, the total phenolic recovery from green peas was increased fivefold (2020–4050 mg/kg dw) [83].

Deep Eutectic Solvents (DESs) and Natural Deep Eutectic Solvents (NADESs), composed of renewable hydrogen bond acceptors (e.g., choline chloride, betaine) and donors (organic acids, sugars, polyols), exhibit low toxicity, high biodegradability, and tunable physicochemical properties [84]. Evidence shows that the choline chloride–glycerol system extracted olive leaf polyphenols more efficiently than ethanol [85] and that betaine–urea NADESs extracted polyphenols from blueberries and green tea with significantly improved antioxidant activity (IC_50_ 28.10 μg/μL vs. ethanol 1615.97 μg/μL) [86].

Technological integration further unlocks potential: UAE-NADES synergistic extraction from apple pomace [87], blueberry pomace [88], and moringa leaves [89] achieved simultaneous improvements in yield and activity. Although DESs/NADESs face challenges such as high viscosity requiring water adjustment [90], and the need for ecotoxicity assessment for some systems, their solvents are recyclable (approximately 50% efficiency retained after 5 cycles) [91], significantly reduce dependence on organic solvents, and form a “green solvent–process intensification” dual-drive model when combined with UAE/MAE/PEF technologies [92]. This integrated strategy not only aligns with green chemistry principles and life cycle assessment requirements but also provides systematic technical support for the sustainable development of plant polyphenols in functional foods, natural preservatives, and precision health interventions.

## 4. Biological Activities and Health-Promoting Mechanisms of CFPs

**Figure 3:**
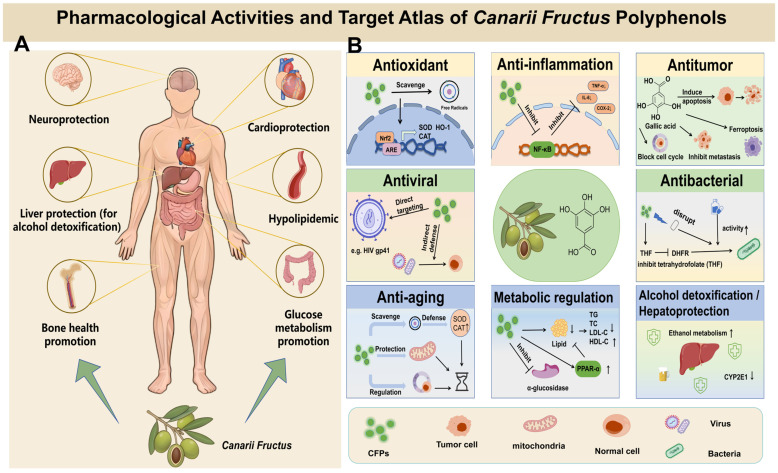
Disease protection and mechanisms of action of CFPs. (**A**) indicates types of disease protection; (**B**) indicates mechanisms of action. Antioxidant: Scavenging free radicals; chelating metal ions; activating the Nrf2/ARE pathway that enhances endogenous antioxidant enzymes. Anti-inflammatory: Inhibiting NF-κB and MAPK pathways; downregulating TNF-α, IL-6, COX-2, and iNOS. Antitumor: Inducing apoptosis; cell cycle arrest; inhibiting metastasis; triggering ferroptosis. Antiviral: Directly targeting HIV gp41, influenza virus NA/PB2; indirectly modulating host anti-inflammatory and antioxidant defenses. Antibacterial: Enhancing drug efficacy. Anti-aging: Protecting mitochondria, scavenging free radicals. Metabolic regulation: Inhibiting α-glucosidase; improving insulin signaling; activating PPAR-α/AMPK. Hepatoprotection and alcohol detoxification: Promoting ethanol metabolism; inhibiting CYP2E1; protecting hepatocytes.

CFPs exhibit a broad spectrum of biological activities characterized by multi-target and multi-pathway regulatory properties. Their health-promoting effects fundamentally arise from antioxidant and anti-inflammatory modulation and extend to antitumor, antiviral, antibacterial, anti-aging, and metabolic regulatory functions. This section synthesizes current mechanistic insights, emphasizing crosstalk among redox balance, inflammatory signaling, metabolic pathways, and host–microbe interactions (Figure 3, Table 5). Before discussing the biological activities of CFPs, it should be noted that the available evidence originates from two distinct sources: studies directly investigating CFP extracts or fractions and studies examining individual polyphenolic constituents identified within CFPs. While CFP-specific studies provide direct evidence of biological efficacy, mechanistic insights are frequently derived from investigations of isolated compounds, including gallic acid, ellagic acid, chlorogenic acid, quercetin, and related phenolics. Therefore, unless otherwise specified, mechanistic interpretations based on individual compounds should be regarded as supportive evidence that may contribute to, but does not definitively explain, the biological effects of CFPs as a whole.

### 4.1. Antioxidant Activity

Oxidative stress describes a pathological condition that emerges when the production of reactive oxygen species (ROS) surpasses the capacity of the cellular antioxidant system, ultimately disturbing redox equilibrium [101]. These phenolics also possess metal-chelating abilities, binding transition metals such as Fe^2+^ and Cu^2+^, thereby suppressing Fenton-type reactions and limiting the generation of highly reactive hydroxyl radicals (•OH) [7,62]. In addition, these polyphenols can chelate transition metal ions such as Fe^2+^ and Cu^2+^, thereby inhibiting Fenton-type reactions and reducing the formation of highly reactive hydroxyl radicals (•OH) [102]. At the level of cellular regulation, polyphenols from CF enhance endogenous antioxidant defenses by modulating the Nrf2 signaling cascade. Through interaction with its inhibitory partner, Kelch-like ECH-associated protein 1 (Keap1), these compounds facilitate the stabilization and nuclear accumulation of Nrf2. Once activated, Nrf2 binds to antioxidant response elements (AREs) and drives the transcription of a suite of phase II detoxifying and antioxidant enzymes, including heme oxygenase-1 (HO-1), superoxide dismutase (SOD), and catalase (CAT). This coordinated response collectively strengthens the cell’s ability to counteract oxidative damage [103,104].

Direct experimental evidence has shown that the ability of CFPs (Chinese white olive phenolic compounds) to directly scavenge free radicals originates from the phenolic hydroxyl groups acting as hydrogen or electron donors to neutralize free radicals. Among them, gallic acid exhibited superior activity to vitamin C, with a half-maximal scavenging concentration (SC_50_) of 0.023, compared to 0.028 for Vc. Ellagic acid and epigallocatechin gallate (EGCG) showed comparable activity to vitamin C, with SC_50_ values of 0.055 and 0.060, respectively, versus 0.038 for Vc [7,62].

Overall, CFPs establish a multi-layered antioxidant defense system through direct free radical scavenging, metal ion chelation, and the systematic upregulation of endogenous antioxidant enzymes such as HO-1, SOD, and CAT via Nrf2/ARE pathway activation. Future research should focus on elucidating the synergistic effects among polyphenolic components and advancing their standardized application in functional foods and products aimed at intervening in oxidative stress-related chronic diseases.

### 4.2. Anti-Inflammatory Effects

Inflammation is a pathological process involving multiple inflammatory cells and cytokines [105]. CF extract can significantly inhibit NO production, and its phenolic components—such as galloyl-bis-HHDP-glucose, gallic acid, and catechin—effectively suppress the secretion of core pro-inflammatory cytokines TNF-α and IL-6 [39]. One of the core anti-inflammatory mechanisms of CFPs is achieved through inhibition of the nuclear factor-κB NF−κB signaling pathway: upon inflammatory stimulation, the inhibitor of κB (IκB) is phosphorylated and degraded by IκB kinase (IKK), enabling activated NF-κB to translocate into the nucleus and initiate transcription of genes including TNF-α, IL-6, cyclooxygenase-2 (COX-2), and inducible nitric oxide synthase (iNOS) [106,107,108].

Meanwhile, gallic acid can prevent the nuclear translocation of NF-κB and signal transducer and activator of transcription 1/3 (STAT1/STAT3), thereby reducing mRNA expression of inflammatory cytokines [109]. Furthermore, certain plant polyphenols can also inhibit phosphorylation of c-Jun N-terminal kinase (JNK) and p38 mitogen-activated protein kinase (p38 MAPK), blocking activator protein-1 (AP-1) activation and consequently downregulating expression of inflammatory mediators such as IL-1β and COX-2 [110,111]. CFPs may possess similar mechanisms.

In summary, CFPs exert systemic anti-inflammatory activity primarily through coordinated inhibition of NF-κB and MAPK signaling pathways, resulting in reduced expression of key inflammatory mediators including TNF-α, IL-6, COX-2, and iNOS. Future studies should focus on in vivo and clinical validation of their anti-inflammatory efficacy, as well as their interactions with the gut microbiota, to support the development of functional foods and therapeutic agents for chronic inflammatory diseases.

### 4.3. Anticancer and Antitumor Properties

Tumorigenesis is a multistep process during which cells acquire a series of mutations that lead to unrestricted growth and proliferation, suppression of cell differentiation, and evasion of cell death [111]. Polyphenolic compounds derived from CF exert antitumor and anticancer effects by modulating multiple signaling pathways and molecular targets.

**Figure 4:**
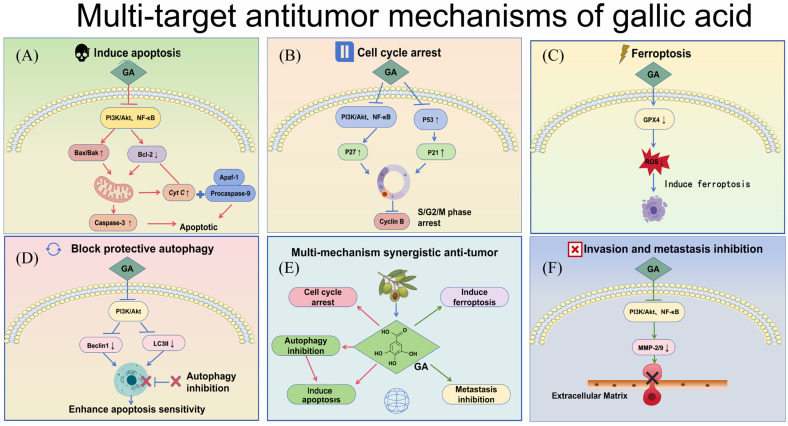
Antitumor mechanisms of GA. (**A**) Induction of apoptosis: GA promotes mitochondrial apoptosis by upregulating pro-apoptotic proteins Bax and Bak while downregulating anti-apoptotic Bcl-2. Concurrent inhibition of PI3K/Akt and NF-κB signaling pathways facilitates activation of the intrinsic apoptotic cascade. (**B**) Cell cycle arrest: GA activates the p53/p21 signaling axis, leading to inhibition of Cdc2/Cyclin B activity and stabilization of p27, thereby inducing arrest at the S and G2/M phases. (**C**) Induction of ferroptosis: GA suppresses glutathione peroxidase 4 (GPX4) activity, triggering iron-dependent lipid peroxidation and ferroptotic cell death, which contributes to overcoming apoptosis resistance. (**D**) Inhibition of invasion and metastasis: GA suppresses PI3K/Akt and NF-κB pathways, resulting in downregulation of matrix metalloproteinases MMP-2 and MMP-9, thereby reducing tumor cell migration and invasion. (**E**) Multi-mechanism synergistic antitumor. (**F**) Suppression of protective autophagy: GA inhibits the expression of autophagy-related proteins Beclin1 and LC3II, thereby blocking cytoprotective autophagy and enhancing apoptotic sensitivity.

Gallic acid (GA), as a representative component, has demonstrated significant antitumor activity in research studies. At the apoptosis induction level, in the intrinsic mitochondrial pathway of apoptosis, when cells are stimulated by death signals such as elevated reactive oxygen species levels or DNA damage, the permeability of the mitochondrial outer membrane increases, promoting the release of cytochrome C (Cyt C) from the mitochondria into the cytoplasm [93]. The released Cyt C binds to apoptotic protease activating factor-1 (Apaf-1) and procaspase-9 to form the apoptosome, which subsequently activates caspase-9 and initiates downstream caspase cascade reactions, ultimately inducing cell apoptosis [94]. GA can activate the aforementioned mitochondrial apoptosis pathway by upregulating pro-apoptotic proteins Bax/Bak and downregulating anti-apoptotic protein B-cell lymphoma-2 (Bcl-2) [95]. Simultaneously, it significantly increases the protein and mRNA expression levels of tumor suppressor genes p53, Bax, and caspase-3, and upregulates the corresponding expression of Cyt C, by inhibiting pro-survival signaling pathways such as phosphoinositide 3-kinase/protein kinase B (PI3K/Akt) and nuclear factor-kappa B (NF-κB). Conversely, the protein and mRNA expression levels of oncogenes Bcl-2, PI3K, Akt, and NF-κB are significantly decreased (Figure 4A) [96].

In terms of cell cycle regulation, GA activates the p53/p21 axis, suppressing cyclin-dependent kinase 1 (Cdc2) and cyclin B activity, thereby arresting tumor cells at the S or G2/M phase. This effect is further reinforced through stabilization of the cyclin-dependent kinase inhibitor p27 (Figure 4B) [112,113,114]. In terms of invasion and metastasis inhibition, gallic acid downregulates matrix metalloproteinase-2/9 (MMP-2/9) expression through inhibition of the PI3K/Akt/NF-κB pathway, thereby weakening the migration and invasion capabilities of tumor cells (Figure 4E) [115,116]. Furthermore, GA can intervene in the autophagy process by inhibiting the expression of autophagy-related protein Beclin1 and microtubule-associated protein 1 light chain 3 II (LC3II), blocking protective autophagy in tumor cells and thereby enhancing their sensitivity to apoptosis induction (Figure 4D) [117] while also inducing ferroptosis by reducing glutathione peroxidase 4 (GPX4) activity, providing a novel strategy for overcoming apoptosis resistance (Figure 4C) [118].

Beyond GA, flavonoids such as kaempferol and quercetin regulate non-coding RNA networks, including microRNAs and long non-coding RNAs, thereby modulating key oncogenic pathways such as p53, Wnt/β-catenin, PI3K/Akt, and Notch [119]. Broader evidence from plant polyphenols suggests that CFPs may exert synergistic antitumor effects via inhibition of PI3K/Akt/mTOR, Wnt/β-catenin, and NF-κB signaling, as well as regulation of the tumor microenvironment (TME) [39,120,121].

Overall, CFPs and their key constituents, such as GA, exert broad-spectrum antitumor effects through integrated regulation of apoptosis, cell cycle arrest, metastasis suppression, autophagy inhibition, and ferroptosis induction, primarily involving the PI3K/Akt, Wnt/β-catenin, and NF-κB pathways. Future studies should prioritize in vivo validation and clinical translation, as well as explore their potential as chemosensitizers and tumor microenvironment modulators.

### 4.4. Antiviral Activity

Viruses are a class of widely distributed organisms that often cause extremely dangerous diseases, as most of them can rapidly proliferate and infect hosts. Pathogenic viruses also frequently mutate, resulting in limited measures for preventing viral transmission and promoting recovery [122]. In contrast, CFP components exhibit multi-target, broad-spectrum antiviral effects.

Regarding human immunodeficiency virus (HIV), the ethyl acetate extract of CF inhibits viral entry by disrupting gp41 six-helix bundle formation, with gallic acid identified as a major active constituent [97]. In addition, CF leaf extracts suppress influenza A virus (IAV) replication in MDCK cells [9]. Mechanistically, isocorilagin inhibits viral release by targeting neuraminidase (NA) activity, whereas methyl brevifolin carboxylate suppresses viral replication by binding to the cap-binding domain of the PB2 subunit of viral RNA polymerase [99]. Beyond direct viral protein targeting, CFPs exert antiviral effects through multiple complementary mechanisms, including suppression of NF-κB-mediated inflammatory responses [123], modulation of host redox homeostasis and gene expression [124], and interference with viral adsorption via competitive binding to viral surface proteins or host receptors [125,126], and regulate miRNA to influence host responses to viral infection [127], collectively exerting comprehensive antiviral effects.

CFPs demonstrate broad-spectrum antiviral potential against viruses such as HIV and IAV through integrated regulation of viral replication, host immune responses, and oxidative stress pathways. Future studies should focus on identifying and characterizing key antiviral constituents and evaluating their potential in overcoming viral drug resistance and modulating host–pathogen interactions.

### 4.5. Antibacterial Effects

Bacterial pathogens can infect a wide range of hosts, including humans, animals, and plants, causing serious health problems. The emergence of antibiotic-resistant strains has increased this risk, making the treatment of bacterial infections more challenging [128]. In contrast, CF and its active components possess broad-spectrum antibacterial effects, potentially offering valuable insights into antibacterial research directions.

GA, as an important active monomer of CFPs, dissipates bacterial proton motive force (PMF) to disrupt the integrity and function of the cytoplasmic membrane, while simultaneously modulating dihydrofolate reductase (DHFR) expression to inhibit tetrahydrofolate (THF) synthesis, thereby affecting bacterial nucleic acid metabolism and enhancing the sensitivity of sulfonamide drugs against multidrug-resistant streptococci [129]. The ethyl acetate extract of CF MIC 39–625 μg/mL can induce morphological changes in Helicobacter pylori (Hp) and inhibit its urease activity IC_50_ 332.90 μg/mL, while downregulating the expression of vacuolating cytotoxin gene A (vacA) and cytotoxin-associated gene A (cagA) [5]. Future research should elucidate their molecular antibacterial targets in depth and explore their application value in novel antibacterial materials or as adjunctive agents in anti-infective therapy.

### 4.6. Anti-Aging Potential

The aging process is accompanied by a chronic inflammatory state with activation of multiple immune pathways [130]. CFP components can exert anti-aging effects through antioxidant, anti-inflammatory, and modulation of aging-related pathways.

In the Caenorhabditis elegans model, CF extract enhances superoxide dismutase (SOD) activity and extends lifespan [131]; in the Drosophila melanogaster model, it also demonstrates lifespan extension effects [132]. Cellular-level studies further elucidate its mechanisms: in D-galactose-induced PC12 cellular senescence models, CFPs dose-dependently alleviate lipid peroxidation, reduce lactate dehydrogenase (LDH) leakage and intracellular reactive oxygen species (ROS) levels, optimally activate SOD, catalase (CAT), and glutathione peroxidase (GSH-Px) activities, improve mitochondrial membrane potential, reduce apoptosis, and promote cell cycle transition from G1 phase to S phase, thereby achieving anti-aging effects by attenuating oxidative damage, maintaining mitochondrial function, and regulating cell cycle progression [62].

These findings demonstrate that CFPs exert anti-aging activity through multidimensional mechanisms, including direct free radical scavenging, enhancement of endogenous antioxidant defense, protection of mitochondrial function, and modulation of aging-related pathways, providing scientific evidence for their development as functional foods or anti-aging lead compounds.

### 4.7. Metabolic Regulatory Effects

Diabetic patients often present with concurrent hyperlipidemia, which together exacerbate vascular damage [133]. CFPs may regulate glucose and lipid metabolism through multi-target and multi-level mechanisms. In terms of hypoglycemic activity, CFPs can attenuate postprandial blood glucose elevation primarily by inhibiting intestinal α-glucosidase, thereby delaying carbohydrate digestion and absorption. Among different extracts, a 70% ethanol extract exhibited the strongest inhibitory effect, showing a reversible non-competitive inhibition mode with an IC_50_ value of 9.914 × 10^−3^ μg/mL in vitro, as reported in an original study [134]; flavonoid components such as quercetin and kaempferol may inhibit this enzyme through similar mechanisms [135,136]. In terms of improving insulin signaling, the ethyl acetate extract of CF enhances insulin sensitivity by inhibiting excessive phosphorylation of hepatic insulin receptor substrate 1 (IRS-1) and enhancing protein kinase B (Akt) phosphorylation while simultaneously stimulating glucose transporter 4 (GLUT4) membrane translocation to increase tissue glucose uptake [31,137].

Regarding lipid metabolism regulation, CFPs exhibited significant lipid-lowering effects in the HepG2 human hepatocellular carcinoma cell model and concentration-dependently reduced intracellular total lipid and triglyceride levels. The mechanisms involved activation of the AMPK signaling pathway (manifested as enhanced AMPK phosphorylation); downregulation of key lipogenic genes, including SREBP-1c, ACC1, FASN, and DGAT2; and upregulation of fatty acid β-oxidation-related genes, including PGC‐1α, PPARα, CPT‐1A, and ACOX1, accompanied by decreased expression of miR-122 and miR-21, suggesting that microRNAs may participate in the regulatory process [138]. Furthermore, their potent antioxidant capacity can reduce lipid peroxidation levels and protect pancreatic β-cells from oxidative stress-induced damage [139].

These systematic mechanisms collectively address the metabolic disorders and oxidative stress underlying diabetes and its complications, highlighting the pleiotropic potential of CFPs in the prevention and management of metabolic syndrome. Future research should focus on the synergistic effects of their multi-component composition and advance product development and clinical application in the management of metabolic syndrome conditions such as diabetes and non-alcoholic fatty liver disease.

### 4.8. Hepatoprotective Effects and Mitigation of Alcohol-Induced Liver Injury

CF and its extracts exert hepatoprotective and alcohol-detoxifying effects through multiple pathways, including antioxidant activity, anti-inflammatory action, metabolic enzyme regulation, and hepatocyte protection. The early compound formula “Olive Hangover-Relieving Beverage” can reduce intoxication rates and increase the awakening/intoxication ratio in acute alcoholic liver injury models [100]. Its flavonoids and polyphenols are the core active components: chlorogenic acid can reduce the generation of toxic metabolite acetaldehyde by inhibiting cytochrome P450 2E1 (CYP2E1) activity and bind to Keap1 to alleviate oxidative stress and liver injury [140]; flavonoid components can protect against ethanol-induced liver injury at the epigenetic level by restoring NRF2 stability and preventing abnormal nuclear accumulation of acetyl-CoA synthetase 2 (ACSS2) and histone H3 acetylation [141]. These studies, ranging from whole compound formulas to active monomers, and from pathological observations to molecular mechanisms, provide scientific evidence for the development of CF as functional beverages or alcohol-detoxifying and hepatoprotective drugs.

### 4.9. Additional Pharmacological Activities

CFPs also possess various traditional and modern pharmacological activities. Regarding bone health, they can increase bone mineral density (BMD) and bone calcium content in rats [142]. Furthermore, CFPs may also ameliorate chronic obstructive pulmonary disease (COPD) by downregulating the mitogen-activated protein kinase (MAPK) signaling pathway to reduce inflammatory mediator release and COX-2 and iNOS expression, among which catechin, epicatechin, and EGCG show strong interactions with core COPD targets and may represent the active constituents [143].

Although substantial progress has been made in elucidating the biological activities of CFPs, the current evidence base remains uneven. Many mechanistic interpretations are inferred from studies of individual polyphenolic constituents rather than CFP extracts themselves (Table 6). While such findings provide valuable insights into potential molecular targets and pathways, direct validation at the extract level remains limited for several biological activities. Future studies integrating phytochemical characterization, bioactivity-guided fractionation, and mechanistic investigations of CFP extracts will be essential for establishing clearer causal relationships.

The health-promoting effects of CFPs are unlikely to be attributable to a single compound. Instead, the coexistence of phenolic acids, hydrolysable tannins, and flavonoids may generate additive or synergistic effects through complementary molecular targets and signaling pathways, a phenomenon widely recognized in polyphenol-rich plant matrices [144,145,146].

## 5. Challenges and Technological Pathways for Future Development

**Figure 5:**
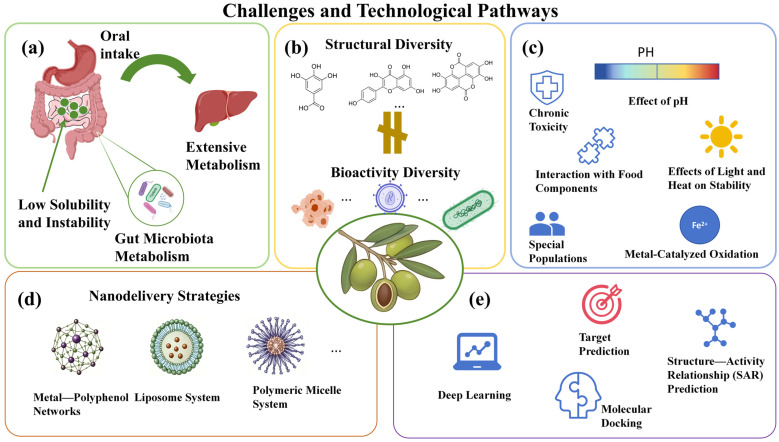
Challenges and technological pathways. (**a**) Low in vivo delivery and transformation efficiency, manifested as poor solubility, complex metabolism, and limited bioavailability. (**b**) Complex structure–activity relationships and unclear multi-component synergistic mechanisms arising from structural diversity and undefined contributions of metabolites. (**c**) Restricted development of functional foods, involving insufficient safety assessment and processing stability issues. (**d**) Enhancing solubility, stability, and targeting through nanodelivery systems (such as metal–polyphenol networks, liposomes, polymeric micelles, and enzymatic modification), thereby improving in vivo bioavailability. (**e**) Integrating artificial intelligence and computational biology techniques (deep learning, molecular docking) to achieve target prediction and multi-component synergistic mechanism elucidation.

Despite the wide range of biological activities demonstrated by CFPs in vitro, their translational potential remains constrained by several critical limitations, including low in vivo bioavailability, inefficient absorption and biotransformation, and incomplete understanding of structure–activity relationships and multi-component synergistic mechanisms, as well as insufficient safety and stability evaluation for functional food development. Collectively, these challenges substantially hinder the progression of CFPs from laboratory research to practical applications. Future development should focus on the integration of complementary technological strategies across multiple scales. On one hand, nanodelivery systems constructed from biocompatible materials, including metal–phenolic networks, lipid-based nanoparticles, polymeric micelles, and enzyme-mediated modification platforms, have shown considerable potential in enhancing solubility, stability, and targeting efficiency, thereby improving systemic bioavailability and in vivo efficacy. On the other hand, artificial intelligence and computational biology approaches, particularly deep learning and molecular docking, are emerging as powerful tools for deciphering complex multi-component systems. These methods enable high-throughput virtual screening, target prediction, and systematic elucidation of synergistic interactions, thereby providing mechanistic insight at a systems level (Figure 5).

### 5.1. Research Challenges

#### 5.1.1. Limitations in in Vivo Delivery and Biotransformation Efficiency

The in vivo bioavailability of CFPs is generally low, which is a common challenge faced by natural polyphenolic compounds. The limiting factors mainly include: physicochemical property constraints, as many phenolic compounds exhibit low water solubility and chemical instability, and are affected by interactions with food matrices and other nutrients [147]; metabolic transformation complexity, as oral polyphenols undergo extensive metabolism by gut microbiota, generating structurally diverse ring-cleavage metabolites whose biological activities may be altered [148,149,150]; and inefficient delivery, as under traditional administration routes, prototype polyphenol compounds have short biological half-lives in vivo, making it difficult to achieve effective concentrations in target organs, while the in vivo distribution and concentration of their active metabolites are challenging to precisely control (Figure 5a) [151].

These inherent pharmacokinetic deficiencies directly lead to significant translational gaps between in vitro and in vivo experimental results. For example, although CF leaf extract demonstrates antiviral activity in Madin–Darby canine kidney (MDCK) cell models, its potential for anti-influenza virus infection in vivo remains to be fully validated [9]. This gap arises because in vitro models typically fail to simulate complex in vivo pharmacokinetic (PK) processes (such as absorption, distribution, metabolism, and excretion) and the microenvironment of multi-organ interactions [152]. Even biological effects observed in simple models such as nematodes (e.g., anti-aging) may be difficult to accurately predict for actual efficacy in humans due to their inability to simulate the complex metabolic networks of mammals [153]. Recent studies have demonstrated that CFPs exhibit significant interactions with the gut microbiota, and their health benefits may depend on intestinal metabolic transformation [154]. This paradox of being “biologically relevant yet non-translatable” is particularly prominent in plant extract research, as release conditions in in vitro experimental designs (such as drug dissolution)—even when similar to in vivo release profiles—may affect actual drug bioavailability by neglecting biological factors (e.g., intestinal absorption, first-pass effects) [155].

Despite the growing interest in CFPs, no standardized quality control system has yet been established. Current studies employ different cultivars, maturity stages, extraction methods, and analytical platforms, resulting in considerable variability in reported phenolic profiles. For example, gallic acid and ellagic acid are consistently identified as the predominant phenolic constituents of CF, whereas chlorogenic acid, corilagin, quercetin, and kaempferol derivatives show varying abundance among studies. Such variability complicates cross-study comparisons and hinders industrial standardization.

Therefore, future efforts should focus on establishing CFP-specific quality markers (Q-markers) and chemical fingerprints based on high-frequency and biologically relevant constituents. The integration of metabolomics and chemometric approaches may facilitate the development of standardized quality evaluation systems for CFP-derived products.

#### 5.1.2. Challenges in Structure–Activity Relationships and Synergistic Mechanisms

Current mechanistic studies primarily focus on individual compounds, particularly gallic acid and ellagic acid. However, CFPs are complex mixtures containing phenolic acids, hydrolysable tannins, and flavonoids. The overall biological activities of CFPs are therefore unlikely to result from a single constituent.

In terms of structure–activity relationship (SAR), although quantitative structure–activity relationship (QSAR) studies indicate that structural features such as ortho-diphenolic hydroxyl groups are positively correlated with antioxidant activity [156], the high structural diversity of CFPs (such as glycosylation and esterification modifications) makes it difficult to establish universal activity prediction models. Studies have shown that different polyphenol monomers exhibit significantly different inhibitory effects on the same target (e.g., tea polyphenols against human pancreatic α-amylase), and their precise structure–activity correlations remain unclear [157]. Meanwhile, CFP substances containing ortho-dihydroxyl structures (such as caffeic acid and quercetin) also demonstrate complex structure-dependent inhibitory effects on specific harmful substances (e.g., heterocyclic amines), and the precise association between active sites and molecular configurations awaits further elucidation [158].

Regarding multi-component synergistic effects, the complex polyphenolic mixtures in CF extracts (such as flavonoids and phenolic acids) may produce additive, synergistic, or even antagonistic effects; however, current research is predominantly based on holistic activity assessments of extracts, with limited understanding of the interactions among individual components and their quantitative contributions to overall efficacy [159,160]. Furthermore, small-molecule metabolites such as phenolic acid derivatives generated by gut microbiota metabolism may exhibit superior or even distinct biological activities compared to prototype compounds, yet their actual contribution proportions and specific mechanisms of action in vivo remain unclear [148,158]. Existing studies excessively rely on activity evaluations of single compounds, neglecting the interactions between polyphenols and other biomolecules within complex dietary matrices—this represents another important reason for the insufficient mechanistic elucidation (Figure 5b). Future work must adopt systems-level approaches to quantify interactions among CFP constituents. This includes: (i) fractionation-guided synergy assays (e.g., testing reconstituted mixtures of GA + quercetin + ellagic acid against α-glucosidase or NF-κB); (ii) multi-omics integration (metabolomics of CFP-treated cells + transcriptomics of target pathways) to map network-level crosstalk; and (iii) AI-driven synergy prediction using deep learning models trained on polyphenol interaction databases. Such efforts will transform CFPs from a “source of actives” into a paradigm of natural synergistic therapeutics.

### 5.2. Technological Pathways for Advancing CFP Utilization

#### 5.2.1. Nanodelivery Strategies to Enhance Polyphenol Bioavailability

A major limitation restricting the clinical application of polyphenolic compounds is their poor bioavailability. Consequently, the development of efficient and safe delivery systems has become a key research focus. Nanodelivery systems based on biocompatible macromolecules, including polysaccharides, proteins, and lipids, have demonstrated considerable potential in improving solubility, stability, and targeting efficiency, thereby enhancing systemic bioavailability of polyphenols [161]. Based on their structural design and assembly mechanisms, these systems can be classified into several major categories.

Metal–phenolic networks based on coordination interactions. Utilizing the abundant phenolic hydroxyl groups in polyphenols to coordinate with metal ions, metal–phenolic networks (MPNs) have emerged as important candidate systems for polyphenol-based nanomedicines, particularly in the field of tumor therapy. Such network structures can effectively load and protect polyphenols, achieving controlled release [162]. Lipid-based nanoparticle systems. Lipid nanoparticles represent one of the most common carriers in drug delivery. For example, encapsulating quercetin, an important polyphenolic component from CF, into liposomes can significantly reduce its metabolic degradation in the liver, improve its pharmacokinetic characteristics, and enhance antitumor functionality (Figure 5d) [163].

Polymer-based micelle systems. Micelles, especially polymer micelles, are widely used to deliver chemotherapeutic drugs and natural products due to their high biocompatibility and excellent drug solubilization capacity, overcoming drug resistance and enhancing therapeutic efficacy. A representative study involves a targeted mixed micelle system for non-small cell lung cancer treatment: researchers employed 1,2-distearoyl-sn-glycero-3-phosphoethanolamine–polyethylene glycol–biotin (DSPE-PEG–biotin) and poly(ethylene glycol) methyl ether methacrylate–poly[(dimethylamino)ethyl acrylate]–poly(ε-caprolactone) (PEGMA-PDMAEA-PCL) to co-assemble quercetin-loaded mixed micelles (Que-MMICs). This system achieved an encapsulation efficiency (EE) as high as 85.7%, and biotin-mediated targeting enhanced cellular uptake efficiency by 1.2-fold. In vitro experiments demonstrated that Que-MMICs exhibited an IC_50_ of 7.83 μg/mL against A549 cells, significantly superior to 44.22 μg/mL for free quercetin, and more effectively induced apoptosis and cell cycle arrest [164]. Another early study loaded quercetin into poly(ethylene glycol)–b-oligo(ε-caprolactone) (PEG-OCL) micelles, achieving approximately 110-fold enhancement in water solubility through introduction of hydrophobic terminal groups, thereby inhibiting tumor progression by arresting the cell cycle at the G2/M phase [165]. Enzymatic modification to enhance solubility. Beyond physical encapsulation, chemical modification of polyphenol molecules represents another strategy. For instance, utilizing tyrosinase for ortho-hydroxylation of polyphenols enables highly selective, high-yield modification without cofactors. Such hydroxylation and glycosylation modifications have been proven to significantly enhance polyphenol water solubility, stability, and biological activity (Figure 5d) [166].

Although nanodelivery systems and AI-assisted approaches have shown considerable promise in the development of plant polyphenols, direct applications to CFPs remain largely unexplored. Current CFP research has mainly focused on phytochemical characterization, metabolomic profiling, extraction optimization, and biological activity evaluation. Recent metabolomic and transcriptomic studies have revealed substantial cultivar-dependent variations in flavonoid and polyphenol composition [10,49], while emerging evidence suggests that CFP bioactivities may involve interactions with the gut microbiota [154]. These findings provide a CFP-specific rationale for future applications of artificial intelligence, systems biology, and advanced delivery technologies. Nevertheless, the lack of CFP-specific formulation studies, pharmacokinetic data, and large-scale datasets currently limits practical implementation. Therefore, these approaches should be regarded as promising future directions rather than established technological solutions for CFPs.

#### 5.2.2. AI-Driven Target Discovery and Mechanistic Analysis of CFPs

Traditional target identification for natural products, particularly for complex systems such as CFPs characterized by structural diversity and complicated mechanisms of action, often faces challenges including time-consuming processes, low throughput, and insufficient resolution of multi-target multi-component (MTMC) synergistic mechanisms. Conventional target identification processes for polyphenolic components are labor-intensive and time-consuming; artificial intelligence (AI) technology can substantially shorten research cycles and enhance the efficiency and systematicity of target discovery [167]. Specifically, deep learning (DL) has been applied to predict polyphenol–protein interactions—for instance, by integrating open ligand–protein interaction experiments with deep learning models, the binding characteristics of polyphenol–protein complexes can be rapidly and accurately predicted, offering novel perspectives for the design of polyphenol-targeted delivery systems [168]. More importantly, AI methodologies can systematically resolve the inherent MTMC therapeutic patterns of traditional Chinese medicine, overcoming the limitations of conventional approaches in elucidating multi-component synergistic mechanisms and thereby enabling a deeper understanding of the pharmacological foundations of complex systems such as CFPs [169].

At the specific computational tool level, molecular docking and molecular dynamics (MD) simulation have become cornerstones for investigating polyphenol–protein interactions. For example, one study employed molecular docking analysis to evaluate the binding energies of several kaempferol glycosides with key inflammatory targets, including nuclear factor-κB (NF-κB), phosphatidylinositol 3-kinase (PI3K), and protein kinase B (Akt), predicting that KGRG (kaempferol-3-O-(2-O-glucopyranosyl-6-O-rhamnopyranosyl)-glucopyranoside) possessed optimal binding potential; subsequent experimental validation confirmed its superior anti-inflammatory activity [170]. However, the structural diversity and binding dynamics of polyphenols impose limitations on scalability and reproducibility for traditional experimental techniques (such as nuclear magnetic resonance (NMR) and mass spectrometry (MS)) as well as the aforementioned computational methods. Emerging deep learning algorithms are reshaping the research paradigm in this field by integrating high-dimensional chemical and biological information to achieve efficient prediction of binding sites, affinity, and even dynamic interaction trajectories (Figure 5e) [171].

Although AI-assisted approaches have not yet been widely applied to CFP research, the rapidly expanding datasets generated from CFP phytochemistry, metabolomics, and bioactivity studies provide a foundation for future implementation. Machine learning algorithms may facilitate the prediction of bioactive compounds, identification of quality markers, optimization of extraction processes, and discovery of structure–activity relationships.

## 6. Potential Applications and Challenges in Food Systems

### 6.1. Application Potential

#### 6.1.1. Application Potential of Food Additives

Among the various biological activities of CFPs, antioxidant activity is the most extensively studied and well-supported, forming the most direct scientific basis for their use as food additives [7]. Consumers’ preference for natural and healthy foods has prompted the food industry to seek natural alternatives to synthetic antioxidants such as Butylated Hydroxyanisole (BHA), Butylated Hydroxytoluene (BHT), and tert-Butylhydroquinone (TBHQ) [172]. High-fat meat products (such as sausages, bacon, and patties) are highly susceptible to lipid oxidation during storage and processing, resulting in rancidity, nutrient degradation, and the formation of harmful aldehydes and ketones [173]. Based on general mechanisms of plant polyphenols, CFPs are reasonably expected to retard fatty acid rancidity in meat systems and reduce Thiobarbituric Acid Reactive Substance (TBARS) levels. Vitamin C (ascorbic acid) is a commonly used color stabilizer and antioxidant synergist in meat products. Polyphenols can interact synergistically with vitamin C [174], regenerating antioxidants or chelating metal ions (Fe^2+^, Cu^2+^), thereby producing a “1 + 1 > 2” enhancement in color protection and lipid oxidation inhibition.

Currently, systematic studies on the direct incorporation of CFPs into meat or lipid systems are lacking. Such studies are needed to evaluate their effects on lipid oxidation, color stability, microbial growth, and sensory attributes.

#### 6.1.2. Opportunities for Functional Beverages and Healthy Snack Products

Polyphenols are not efficiently absorbed in the small intestine and are mainly metabolized by the gut microbiota. CFPs have been reported to increase the abundance of beneficial bacteria such as Akkermansia, suggesting potential applications in prebiotic or gut-modulating foods [175]. More recently, CFPs were shown to interact extensively with human gut microbiota and may contribute to intestinal homeostasis and inflammatory bowel disease management through microbiota-mediated metabolism. These findings support the development of CFP-enriched functional foods targeting gut health and personalized nutrition applications [154].

Recent metabolomic studies have revealed substantial cultivar-dependent differences in flavonoids and polyphenols, which directly influence flavor characteristics, astringency, and consumer acceptability. Fresh edible cultivars exhibit reduced flavonoid-associated astringency and improved sensory quality, providing opportunities for developing CFP-enriched beverages, fermented products, fruit-based snacks, and low-sugar functional foods [19]. In addition, cooking-mimicking extraction studies have demonstrated that bioactive compounds remain functionally relevant after food processing, suggesting the feasibility of incorporating CFPs into commonly consumed food products rather than relying solely on pharmaceutical-type preparations [176].

#### 6.1.3. Challenges for Food Applications

The commercial adoption of CFPs in food systems is substantially constrained by a confluence of intrinsic chemical, physiological, and sensory limitations, alongside critical gaps in quality assurance and delivery technologies. From a processing standpoint, CFPs exhibit pronounced chemical instability, as their phenolic constituents are highly susceptible to oxidative degradation, thermal breakdown, and non-specific interactions with proteins and polysaccharides during manufacturing [177], which collectively attenuate their functional bioactivity—a challenge severely exacerbated by the lack of standardization, wherein wide variations in cultivar genetics, fruit maturity, and post-harvest processing generate inconsistent phenolic profiles that complicate rigorous quality control and product reproducibility [19,20]. Concurrently, the organoleptic profile poses a formidable obstacle to consumer acceptance, as the pronounced bitterness and astringency inherent to its tannin-rich composition necessitate extensive formulation optimization and flavor-masking strategies, while physiologically, the major bioactive markers—particularly hydrolysable tannins and ellagic acid derivatives—suffer from poor bioavailability due to limited intestinal absorption and extensive first-pass metabolism, critically undermining their potential in vivo efficacy [20]. Although advanced encapsulation and microencapsulation technologies present a promising avenue for enhancing stability, enabling controlled release, and improving bioaccessibility within complex food matrices, their practical validation remains insufficiently explored for CFP-specific applications, marking a pivotal research gap that must be addressed to transition these promising bioactives from bench-scale findings to industrial-scale utilization.

Overall, although CFPs exhibit promising functional properties in food-related contexts, the current body of evidence remains limited in scale and scope, and most advanced application concepts are extrapolated from general polyphenol research rather than CFP-specific food system validation. To facilitate comparison of different application scenarios, a summary table is included that systematically presents the potential food applications of CFPs along with their advantages, limitations, and current stage of development. This structured overview highlights that while CFPs show promising functional properties, most applications remain at the experimental or early development stage, with only limited traditional food uses being well established (Table 7).

### 6.2. Challenges in Processing and Storage Stability

Interactions between polyphenols and other food components (such as proteins and polysaccharides) may lead to unexpected outcomes. For instance, the binding of polyphenols to dietary proteins may not only reduce the digestibility of the proteins themselves but also simultaneously affect the bioaccessibility of both amino acids and polyphenols; such nutrient–nutrient interference has potential implications for the final nutritional efficacy and safety of products [178]. Studies have demonstrated significant differences in the extent of non-covalent interactions between various polyphenols (such as quercetin, resveratrol, and curcumin) and rice proteins, and these differences in binding capacity may further influence their functional properties in food systems (e.g., as emulsifiers), implying complex synergistic or antagonistic effects among multi-component systems [179].

On the other hand, the chemical stability of polyphenols during food processing and storage represents another major technical bottleneck for their application. Their stability is highly dependent on environmental conditions, particularly pH and ionic strength. For example, anthocyanins exhibit color stability under acidic conditions but are prone to structural changes and fading in neutral to alkaline environments [74]; meanwhile, elevated environmental pH leads to deprotonation of polyphenolic hydroxyl groups, enhancing antioxidant activity and reflecting the high environmental sensitivity of their activity [180]. Metal ions in the environment (such as Fe^3+^) can promote polyphenol oxidation by catalyzing the Fenton reaction [102], while changes in ionic strength also significantly affect the solubility and dispersion state of polyphenol–protein complexes, consequently influencing their homogeneity and functional performance in final products (Figure 5c) [181].

### 6.3. Safety and Regulatory Considerations

At present, comprehensive safety assessment frameworks for CFPs and other plant polyphenols remain inadequate, particularly regarding long-term interactions within complex food matrices [103,182,183]. Insufficient information is available on long-term or high-dose intake, use in sensitive populations (such as individuals prone to allergies), and potential risks when coexisting with other food components such as Titanium Dioxide Nanoparticles (TiO_2_ NPs) [10].

The legal use of food ingredients depends on the regulatory frameworks of different countries and regions. CF has been classified as a “dual-use food–medicine substance” by the National Health Commission of China [4], allowing whole fruits or minimally processed forms (such as dried fruits or powders) to be used as regular food ingredients. However, if high-purity or high-concentration polyphenol extracts are prepared using modern technologies (e.g., solvent extraction or chromatographic purification) for use as functional additives, the regulatory pathway may become considerably more complex. Additional safety evidence may be required to demonstrate equivalence to traditional whole-fruit safety, or a new food additive approval process may be necessary. However, in the European Union, CFP-derived products would be regulated under the Novel Food Regulation (EU) 2015/2283, which requires comprehensive safety evaluation, including compositional characterization, toxicological assessment, and evidence of safe use. In the United States, CFP ingredients intended for food applications must comply with the Generally Recognized as Safe (GRAS) framework or food additive regulations, both of which require robust safety data and exposure assessments. Despite the growing evidence of bioactivity, current CFP research remains primarily focused on phytochemical characterization and in vitro/in vivo efficacy, while systematic toxicological evaluation and human safety data are still limited. Therefore, future studies addressing safety profiling, standardized intake levels, and regulatory toxicology will be essential for the international commercialization of CFP-based functional foods.

No commercial food products have been identified that explicitly use *C. Fructus* extracts as core natural antioxidants. Existing local products (such as candied fruits or preserved fruits) employ whole fruits rather than standardized polyphenolic ingredients. Likewise, no authorized patents have been found concerning chemical derivatives, structural modifications, or stability-enhancing applications of CFPs [10]. The simultaneous absence of products and patents indicates that the food applications of CFPs remain at an early academic exploratory stage, with industrial development yet to emerge.

## 7. Key Findings and Future Perspectives

CA is a medicinal and edible fruit tree with a long history of cultivation in southern China, where its fruit, CF, has been traditionally used for both nutritional and therapeutic purposes. In recent years, growing attention has been directed toward the bioactive constituents of CF, particularly its polyphenolic compounds, which exhibit a broad spectrum of biological activities. This review systematically summarizes the chemical composition, extraction and purification strategies, and pharmacological effects of CFPs, with an emphasis on antioxidant, anti-inflammatory, antitumor, antiviral, antibacterial, anti-aging, metabolic regulatory, and hepatoprotective functions.

The key findings and future perspectives are summarized as follows. (1) Chemical diversity and structural characteristics: Approximately 30 polyphenolic compounds have been identified from CF, including gallic acid, ellagic acid, chlorogenic acid, quercetin, kaempferol, and luteolin. These components exhibit diverse structural features, such as ortho-diphenolic hydroxyl groups and glycosylation modifications, which are closely associated with their bioactivities. (2) Extraction and purification technologies: Traditional solvent extraction remains operationally simple but suffers from long extraction times and low efficiency. Emerging techniques, including UAE, MAE, and UMAE, significantly improve extraction yield and reduce processing time. Among them, UMAE achieves the highest yield (6.33%) within only 4.4 min, representing a promising direction for efficient CFP extraction. (3) Biological activities and mechanisms: CFPs exert multi-target and multi-pathway regulatory effects. Their antioxidant activity is mediated by direct free radical scavenging, metal ion chelation, and activation of the Nrf2/ARE signaling pathway. Their anti-inflammatory effects are primarily achieved through inhibition of the NF-κB and MAPK pathways, downregulating TNF-α, IL-6, COX-2, and iNOS. Antitumor activities involve induction of apoptosis, cell cycle arrest, ferroptosis, and inhibition of metastasis via the PI3K/Akt, Wnt/β-catenin, and p53 pathways. Furthermore, CFPs exhibit antiviral (HIV, influenza A), antibacterial (including Helicobacter pylori), anti-aging, and metabolic regulatory effects, such as improving insulin sensitivity and activating AMPK-mediated lipid metabolism. (4) Challenges in application: Despite their promising bioactivities, the in vivo bioavailability of CFPs remains low due to poor water solubility, chemical instability, extensive metabolism by gut microbiota, and short biological half-lives. Additionally, the structure–activity relationships and multi-component synergistic mechanisms are not yet fully understood, and systematic safety assessments for long-term use in food systems are lacking.

Although significant progress has been made in the study of CFPs, several key scientific issues remain to be addressed. Future research directions can be summarized as follows. (1) The establishment of standardized chemical markers and quality control systems remains an urgent priority. At present, CFP composition varies significantly depending on cultivar, maturity stage, and processing conditions, resulting in poor comparability across studies and limited industrial applicability. Therefore, future studies should focus on constructing reliable chemical fingerprints and identifying stable marker compounds for quality evaluation. (2) The structure–activity relationships (SARs) of CFP constituents require further systematic investigation. Although major compounds such as gallic acid, ellagic acid, and flavonoids have been identified, the influence of specific structural features, including galloylation, glycosylation, and hydroxyl substitution patterns, on biological activity remains insufficiently clarified. (3) The multi-component synergistic mechanisms of CFPs have not been fully elucidated. Current studies mainly focus on individual compounds, while the potential synergistic, additive, or antagonistic interactions among phenolic acids, hydrolysable tannins, and flavonoids remain largely unexplored, limiting a comprehensive understanding of their holistic bioactivity. (4) The bioavailability and in vivo metabolic fate of CFPs still require further clarification. Due to poor water solubility, chemical instability, and extensive gut microbiota-mediated transformation, the systemic exposure of CFP bioactives is likely limited, and more studies integrating pharmacokinetics and metabolomics are needed. (5) Systematic safety evaluation and clinical evidence are still insufficient. Although CFPs have a long history of dietary consumption, long-term toxicity studies, dose–response relationships, and human intervention trials remain scarce, which restricts their regulatory acceptance in functional food applications.

From a technological and translational perspective, the development of CFP-based functional ingredients is currently at an early stage, corresponding approximately to a technology readiness level (TRL) of 2–4. Most existing studies are limited to laboratory-scale extraction optimization, in vitro biological assays, and preliminary animal experiments, while industrial-scale production and product standardization are still lacking. In terms of commercialization prospects, CFPs demonstrate considerable potential as natural functional ingredients in antioxidant-rich foods, gut health-oriented products, and plant-based nutraceutical formulations. However, their successful commercialization will depend on overcoming several key bottlenecks, including compositional variability, insufficient process scalability, lack of standardized extraction and purification technologies, and regulatory uncertainties.

Looking forward, several opportunities can be envisioned for future development. Green and scalable extraction technologies, such as enzyme-assisted extraction and pulsed electric field-assisted systems, should be further explored to improve efficiency and sustainability. Advanced formulation strategies, including nanodelivery systems, lipid-based carriers, and polymeric encapsulation, may enhance the stability and bioavailability of CFPs. In addition, the integration of artificial intelligence, chemometrics, and multi-omics technologies is expected to facilitate quality control, functional prediction, and mechanism elucidation, thereby accelerating industrial translation. Finally, the establishment of comprehensive safety assessments and regulatory frameworks will be essential for the global commercialization of CFP-based functional products.

In conclusion, CFPs represent a promising natural resource with significant potential for functional food and nutraceutical applications. However, substantial efforts are still required to bridge the gap between laboratory research and industrial application through advances in standardization, mechanistic understanding, bioavailability improvement, and process engineering.

## Figures and Tables

**Table 1:** Comparison of previous reviews and the present review.

Topic	Existing Reviews	Limitation
*CA*	Traditional uses and phytochemistry	Lack of CFP-focused discussion
Plant polyphenols	General mechanisms	No CFP-specific analysis
Functional food polyphenols	Food applications	No discussion of CA
Present review	CFP-centered integrated review	Addresses all above gaps

**Table 2:** Main polyphenol compounds in CF.

Component Name	Structure	Relative Abundance/Content	Pharmacological Activity	References
gallic acid	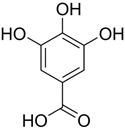	~38.8% of total identified phenolics; detection frequency (100%)	Antioxidant, cardioprotection, neuroprotection, anti-diabetic, antitumor, antibacterial and anti-inflammatory	[26,27]
protocatechuic acid	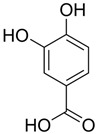	Detection frequency (100%)	Antioxidant, neuroprotection, antibacterial, inhibit platelet aggregation	[28,29]
salicylic acid	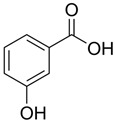	Detection frequency (minor)	Anti-inflammatory, cardioprotection, antitumor	[30,31]
4-hydroxybenzoic acid	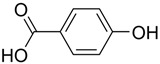	Detection frequency (minor)	Antioxidant, antibacterial and anti-inflammatory	[32]
sinapic acid	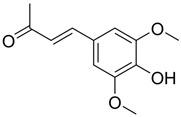	Detection frequency (moderate)	Antioxidant, antitumor, antibacterial, neuroprotection	[26,33]
caffeic acid	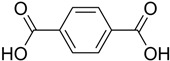	Moderate	Antioxidant, anti-diabetic, antiviral, antibacterial, anticancer, anti-inflammatory	[26,34]
3,3′-di-*O*-methylellagic acid	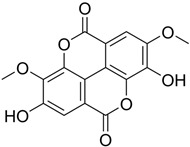	Detection frequency (minor)	——	[26]
digallic acid	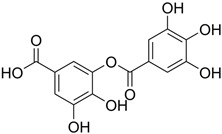	Detection frequency (predominant)	——	[31]
3-*O*-galloyl quinic acid	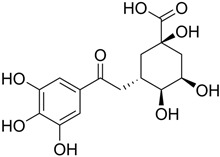	Detection frequency (moderate)	——	[26]
vanillic acid	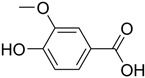	Detection frequency (predominant)	Antioxidant, anti-diabetic, nephroprotection, anti-hepatic fibrosis	[35,36]
3-*O*-galloyl quinic acid butyl ester	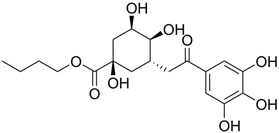	Detection frequency (minor)	——	[26]
brevifolin carboxylic acid	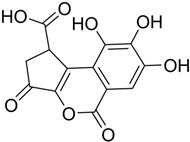	Detection frequency (major)	——	[29]
ellagic acid	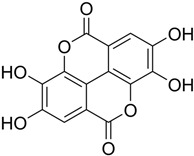	~14.3% of total identified phenolics; detection frequency (100%)	Antioxidant, antiviral, antibacterial, anti-inflammatory, antitumor, hypolipidemic, hypoglycemic, hepatoprotection, anti-osteoporotic, anti-atherogenic, immunomodulation	[26,37]
chlorogenic acid	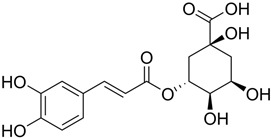	Major phenolic acid; detection frequency (100%)	Antioxidant, antibacterial and anti-inflammatory, hypolipidemic, hypoglycemic	[26,38]
isocorilagin	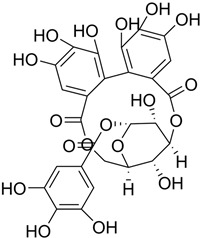	Detection frequency (predominant)	——	[31]
methyl gallate	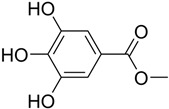	Detection frequency (major)	——	[29]
ethyl gallate	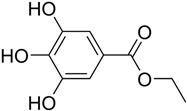	Detection frequency (major)	——	[26]
propyl gallate	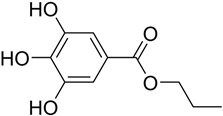	Detection frequency (minor)	——	[26]
methyl brevifolincarboxylate	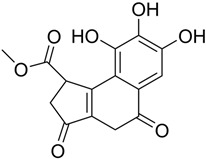	Detection frequency (minor)	——	[26]
octyl gallate	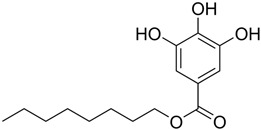	Detection frequency (minor)	——	[26]
ferulic acid	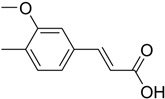	Detection frequency (predominant)	——	[36]
*trans*-cinnamic acid	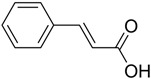	Detection frequency (minor)	——	[36]
procyanidin B1	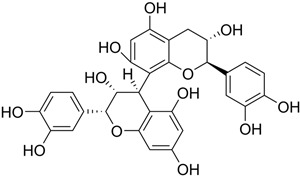	Detection frequency (minor)	——	[39]
epigallocatechin gallate	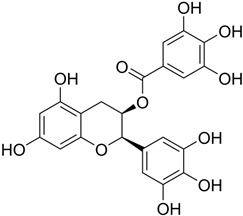	Detection frequency (moderate)	——	[7]
hyperin	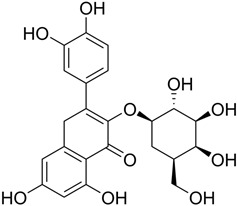	Detection frequency (predominant)	Anticancer, anti-inflammatory, antibacterial, antiviral, antidepressant, organ protective	[29,40]
rutin	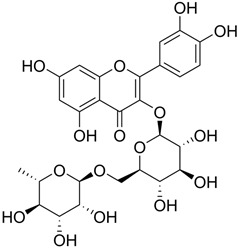	Detection frequency (100%)	Antioxidant, anti-inflammatory, inhibit platelet aggregation	[26,41]
amentoflavone	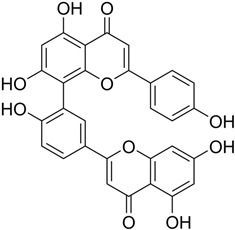	Detection frequency (moderate)	——	[42]
epicatechin	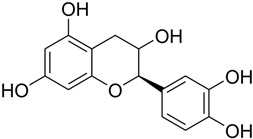	Detection frequency (predominant)	Antioxidant, anti-inflammatory, neuroprotection	[26,43]
kaempferol	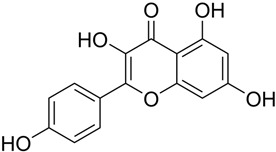	Detection frequency (predominant)	Anti-inflammatory, antibacterial, anticancer	[26,44]
quercetin	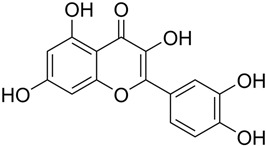	Detection frequency (predominant)	Antitumor, antioxidant, anti-inflammatory, vasculoprotection	[36,45]
luteolin	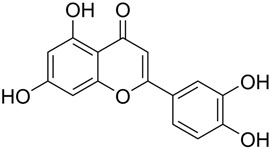	Detection frequency (predominant)	Antioxidant, anti-inflammatory, anticancer, regulate metabolism, neuroprotection	[39,46]
kaempferol-3-O-β-D-glucoside	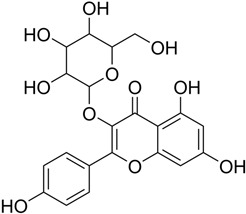	Detection frequency (predominant)	——	[36]

(1) Because quantitative concentration data are unavailable or not directly comparable for many CFP constituents, relative abundance classifications were assigned based on compound detection frequency across independent phytochemical studies and available compositional evidence. Detection frequency was calculated as the proportion of studies reporting a given compound among all phytochemical investigations of CFPs. Compounds detected in ≥75% of studies were classified as predominant, 50–74% as major, 25–49% as moderate, and <25% as minor. These classifications reflect the relative prevalence and consistency of occurrence of CFP constituents in the literature rather than their absolute concentration in the fruit. (2) The biological activities summarized in this table are primarily derived from studies of the corresponding isolated compounds reported in the literature and do not necessarily represent activities directly validated for CFP extracts. These data are included to illustrate the potential functional relevance of individual CFP constituents. “——” indicates that no relevant studies have been reported for the corresponding biological activity.

**Table 3:** Solvent extraction method of polyphenols in CF.

Materials	Temperature/°C	Solvent	Time/min	Solid–Liquid Ratio	Times	Extraction Results	References
Fruit	20~30	60–70% aqueous acetone	60	1:20	1	16.9%	[26]
Fruit	64.4	78.2% methyl alcohol	444	1:20	1	280 mg/g	[58]
Fruit	40	50% ethanol	120	1:30	2	32.13%	[59]
Fruit	70	60% ethanol	120	1:20	2	5.8%	[60]

**Table 4:** Ultrasonic-assisted extraction method of polyphenols in CF.

Materials	Solvent	Power/W	Time/min	Solid–Liquid Ratio	Times	Extraction Results	References
Fruit	30% ethanol	—	40	1:30	1	23 mg/g	[63]
Fruit	40% ethanol	40	45	1:20	3	3.4%	[64]
Fruit	60~70% aqueous acetone	80	25	1:20	1	17.8%	[26]
Fruit	50% ethanol	186	22	1:14	1	5.349%	[62]

**Table 5:** Summary of the biological activities and health-promoting effects of CFPs.

Biological Activity	Experimental Model	Tested Material	Concentration/Dose	Major Outcomes	References
Antioxidant	DPPH, ABTS	Polyphenolic monomer compounds extracted from CF	The specific concentration was not detailed.	Activate the Nrf2/ARE pathway to upregulate the expression of endogenous antioxidant enzymes such as HO-1, SOD, and CAT.	[7]
Anti-inflammatory	RAW264.7 mouse macrophages stimulated with LPS (1 μg/mL)	Free phenolic and bound phenolic extracts from three olive varieties	The specific concentrations used in the anti-inflammatory experiment were not explicitly specified in the article. It only specified that an appropriate concentration was selected based on cell viability assays (no toxicity observed within the range of 25–200 μg TPC/mL) (TPC: total phenolic content).	It significantly inhibits NO, TNF-α, and IL-6 production, and the conjugated phenol exhibits stronger anti-inflammatory potential in certain varieties.	[39]
Anticancer and antitumor	Various cancer cell lines, mouse models	Gallic acid	The dosage was not specified.	Apoptosis promotion: Activate mitochondrial pathway (upregulate Bax/Bak, downregulate Bcl-2, increase Cyt C and caspase-9/3, upregulate p53); inhibit PI3K/Akt/NF-κB. Cell cycle arrest: Activate p53/p21.	[93,94,95,96]
Antiviral	MDCK cells (infected with influenza A virus IAV), HIV pseudovirus/target protein model	Leaf extract of CF, isocorilagin, methyl brevifolin carboxylate, gallic acid	Leaf extract: Inhibits IAV replication (EC50 not provided). Isocorilagin: targets neuraminidase (NA). Methyl brevifolin carboxylate: targets the PB2 subunit.	Inhibits the formation of the HIV gp41 hexagonal helix bundle (preventing viral entry); suppresses IAV release (isocorilagin) or replication (methyl brevifolin carboxylate). Indirect mechanisms include inhibition of NF-κB-mediated inflammatory responses, regulation of host redox homeostasis, and interference with viral adsorption.	[97,98,99]
Antibacterial	Helicobacter pyloriin vitro culture	Ethyl acetate extract of CF	MIC: 39–625 μg/mL, urease IC_50_: 332.90 μg/mL.	Induces morphological changes in Hp. Inhibits urease activity. Downregulates the expression of virulence genes vacA and cagA. GA disrupts the bacterial proton motive force (PMF), inhibits dihydrofolate reductase (DHFR), and enhances the efficacy of sulfonamide drugs.	[5]
Anti-aging	D-galactose induce senescence in PC12 cells, C. elegans and D.melanogaster	CFPs	PC12: Dose-dependent administration in nematodes/fruit flies via feed addition.	Reduces oxidative damage, protects mitochondrial function, promotes G1/S transition, and extends lifespan in model organisms.	[62]
Metabolic regulatory	In vitro inhibition assay of STZ-induced diabetes in HepG2 hepatocellular carcinoma cells (lipid metabolism) using α-glucosidase	70% ethanol extract; ethyl acetate extract; monomers: quercetin, kaempferol	Alpha-glucosidase IC50:9.914 × 10^−3^ μg/mL (70% ethanol extract). Rats: Oral administration (dose not specified).	Lowers blood glucose by inhibiting α-glucosidase and enhancing insulin sensitivity via reducing IRS-1 overphosphorylation, increasing Akt phosphorylation, and promoting GLUT4 translocation.	[31]
Liver-protecting and alcohol-relieving	Acute alcoholic liver injury mouse/rat model	Olive wine alcohol-relieving formula (containing CF)	The dosage was not specified.	Chloroacetic acid inhibits CYP2E1, thereby reducing acetaldehyde production; in combination with Keap1, it alleviates oxidative stress.	[100]

**Table 6:** Evidence strength supporting the proposed biological mechanisms of CFPs.

Biological Activity	Mechanism of Action	Evidence Source	Evidence Strength
Antioxidant	Nrf2/ARE, Keap1-Nrf2 interaction	CFPs + individual CFP constituents (mainly gallic acid/ellagic acid)	Strong [7,62]
Anti-inflammatory	NF-κB (suppress the secretion of core pro-inflammatory cytokines TNF-α and IL-6)	CFPs + individual CFP constituents	Strong [39,109]
Anti-inflammatory	MAPK	Limited CFP-specific evidence	Limited [33]
Anticancer and antitumor properties	Induction of apoptosis, cell cycle arrest, induction of ferroptosis, inhibition of invasion and metastasis, suppression of protective autophagy	Primarily CFP constituent studies (Mainly gallic acid)	Moderate [8,94,95,96,113,116]
Antiviral	HIV and IAV	CFPs (mainly gallic acid) + constituent studies	Strong [97,99]
Antibacterial	Dissipates bacterial proton motive force (PMF) to disrupt the integrity and function of the cytoplasmic membrane	Ethyl acetate extract of CF + primarily CFP constituent studies (mainly gallic acid)	Moderate [5,129]
Anti-aging	Antioxidant, anti-inflammatory	CFPs + individual CFP constituents	Strong [62,131]
Metabolic regulation	Inhibits intestinal α-glucosidase, AMPK	CFPs + individual CFP constituents	Strong [31,137,139]
Hepatoprotective effects and mitigation of alcohol-induced liver injury	Antioxidant, anti-inflammatory	Individual CFP constituents	Limited–moderate [100]

**Table 7:** Potential food applications of CFPs: advantages, limitations, and development status.

Food Application	CFP Material	Advantages	Limitations	Development Status
Functional beverages	CFP extract	High antioxidant capacity; natural bioactive source	Astringency; instability under processing	Experimental use
Preserved fruits and snacks	Whole fruit	Natural preservation; antimicrobial activity	Sensory bitterness; compositional variability	Traditional use/early development
Natural food preservatives	CFP extract	Lipid oxidation inhibition; shelf-life extension	Interaction with food matrix proteins/lipids	Theoretical Stage
Gut health functional foods	CFP	Modulation of gut microbiota; anti-inflammatory potential	Low bioavailability; limited human evidence	Laboratory scale
Fermented foods	CFP-added substrates	Enhanced bioactivity during fermentation	Process-dependent degradation of phenolics	Laboratory scale
Nutraceutical ingredients	Purified fractions	High bioactivity; standardized composition potential	Costly purification; scalability issues	Early development stage

## Data Availability

No new data was created or analyzed in this study.

## Abbreviations

CFP	*Canarii Fructus* polyphenols
CA	*Canarium album*
CF	*Canarii Fructus*
UAE	Ultrasound-assisted extraction
MAE	Microwave-assisted extraction
UMAE	Ultrasound–Microwave Synergistic Extraction
DESs	Deep Eutectic Solvents
NADESs	Natural Deep Eutectic Solvents
ROS	Reactive oxygen species
Nrf2/ARE	Nuclear factor erythroid 2-related factor 2/antioxidant response element
NF-κB	Nuclear factor kappa-light-chain-enhancer of activated B cells
PI3K/Akt	Phosphoinositide 3-kinase/protein kinase B
AMPK	AMP-activated protein kinase
MAPK	Mitogen-activated protein kinase
JNK	c-Jun N-terminal kinase
p38 MAPK	p38 mitogen-activated protein kinase
AP-1	Activator protein-1
TNF-α	Tumor Necrosis Factor-alpha
IL-6	Interleukin-6
COX-2	Cyclooxygenase-2
iNOS	Inducible nitric oxide synthase
MMP-2/9	Matrix metalloproteinase-2/9
GPX4	Glutathione peroxidase 4
SOD	Superoxide dismutase
CAT	Catalase
IC_50_	Half maximal inhibitory concentration
HO-1	Heme oxygenase-1
GSH-Px	Glutathione peroxidase
IRS-1	Insulin receptor substrate 1
GLUT4	Glucose transporter 4
SREBP-1c	Sterol Regulatory Element-Binding Protein-1c
ACC1	Acetyl-CoA Carboxylase 1
FASN	Fatty Acid Synthase
DGAT2	Diacylglycerol O-Acyltransferase 2
PGC−1α	Peroxisome Proliferator-Activated Receptor Gamma Coactivator 1-alpha
PPARα	Peroxisome Proliferator-Activated Receptor alpha
CPT−1A	Carnitine Palmitoyltransferase 1A
ACOX1	Acyl-CoA Oxidase 1
Keap1	Kelch-like ECH-associated protein 1
Apaf-1	Apoptotic protease activating factor-1
Cyt C	Cytochrome C
Bcl-2	B-cell lymphoma-2
Bax/Bak	BCL2-Associated X Protein/BCL2 Antagonist/Killer
p53	Tumor protein p53
p21	Cyclin-dependent kinase inhibitor 1A
p27	Cyclin-dependent kinase inhibitor 1B
LC3II	Microtubule-associated protein 1 light chain 3 II
Beclin1	Beclin-1
CYP2E1	Cytochrome P450 2E1
EGCG	Epigallocatechin gallate
NA	Neuraminidase
PB2	Polymerase Basic Protein 2
GA	Gallic acid
IAV	Influenza A virus
Hp	Helicobacter pylori
TBARSs	Thiobarbituric Acid Reactive Substances
Q-marker	Quality marker
TRL	Technology readiness level
GRAS	Generally Recognized as Safe
